# In Silico Evaluation of the Biopharmaceutical and Pharmacokinetic Behavior of Metronidazole from Coated Colonic Release Matrix Tablets †

**DOI:** 10.3390/pharmaceutics17050647

**Published:** 2025-05-14

**Authors:** Roberto Arévalo-Pérez, Cristina Maderuelo, José M. Lanao

**Affiliations:** 1 Department of Pharmaceutical Sciences, Faculty of Pharmacy, University of Salamanca, 37007 Salamanca, Spain; 2 Institute of Biomedical Research of Salamanca (IBSAL), 37007 Salamanca, Spain

**Keywords:** SIMCYP, physiologically based pharmacokinetic modeling (PBPK), physiologically based biopharmaceutics modeling (PBBM), advanced dissolution absorption model (ADAM), biopharmaceutical simulations, pharmacokinetics, in silico model, drug release, colon drug delivery systems, enteric coating, metronidazole

## Abstract

**Background:** Physiologically based biopharmaceutics modeling (PBBM) models can help to predict drug release and in vivo absorption behaviors. Colon drug delivery systems have gained interest over the past few years due to the advantages they provide in treating certain diseases in a local way. The objectives of this work were to simulate the biopharmaceutical and pharmacokinetic behavior of metronidazole hydrophilic matrices coated with different enteric polymers and to highlight the factors with a significant impact on the simulated pharmacokinetic parameters. **Methods**: Physicochemical properties of metronidazole were introduced into Simcyp^®^ simulator platform, and the Advanced Dissolution Absorption Model (ADAM) was employed to simulate the in vivo intestinal absorption and colonic concentrations of metronidazole using a PBBM model. A Kruskal–Wallis test was carried out in order to determine which one of the factors studied has a statistically significant impact on the pharmacokinetic parameters (AUC, C_max_, and T_max_) simulated. **Results:** Enteric-coated matrix tablets are capable of avoiding metronidazole absorption in the small intestine and releasing it in the colonic region. The release and absorption rates of metronidazole depend largely on the percentage of weight gain of the coating and also on the coating agent. Coated tablets with a time-dependent coating show less variability. **Conclusions:** PBBM models can help predict the release from drug delivery systems and the pharmacokinetics in vivo of metronidazole from data obtained in vitro, although complementary in vivo studies should be needed.

## 1. Introduction

In silico simulation tools can help to predict drug release and in vivo absorption behaviors from the results of in vitro tests, and their use has gained relevance in recent years in the development of new formulations, being increasingly requested in the registration processes of new drug products by different regulatory agencies [1]. Many of these tools are based on what are known as “physiologically based pharmacokinetic (PBPK) models”. These are mechanistic models that combine the physical–chemical information of the active substance and the experimental data generated in vitro during the preclinical phases of development with demographic data, physiological, and genetic information from different patient populations to predict parameters and in vivo pharmacokinetic profiles [1]. Although most of the PBPK models focus on the evaluation of potential drug–drug interactions, these tools can also integrate information related to the pharmaceutical formulation and the gastrointestinal (GI) system dynamics to predict oral drug absorption, which, among other preclinical data on drug disposition, can be used to predict serum concentrations versus time [2]. These applications are useful throughout drug product development and are known as physiologically based biopharmaceutics modeling (PBBM).

In silico models facilitate the efficient prediction of biopharmaceutical and pharmacokinetic behavior, as well as the physical–chemical and physiological factors associated with the release, absorption, and disposition of drugs from different pharmaceutical forms, reducing the need for experimental studies. These types of models have some disadvantages deriving from the actual predictive capacity of the model associated with the complexity of physiological systems and consequently need to be validated with data from in vitro and in vivo experiments.

The Simcyp^®^ population-based platform is a mechanistic PBPK modeling tool that combines the demographic, physiological, and genetic information of different patient populations with physico-chemical and preclinical drug experimental data obtained in vitro to predict in vivo pharmacokinetics (PK) parameters and profiles [3]. The Advanced Dissolution Absorption Model (ADAM) implemented in Simcyp^®^ takes into account the variability of different physiological processes affecting oral bioavailability, such as gastric emptying, GI transit times, GI pH, bile salt concentrations, fluid dynamics, intestinal blood flow, fasted-fed status, and diverse enzymes and gut transporters [4].

Colon drug delivery systems have gained interest over the past few years due to the advantages they provide in treating certain diseases in the colon. Treating the disease in a local way avoids the systemic absorption of the drug and thus decreases the potential side effects associated with the treatment [5,6]. The development of hydrophilic matrices is one of the most widely used technological strategies when it comes to achieving drug release in the colon, thanks to the robustness of the method for obtaining tablets, as well as the safety, efficacy, and acceptance by patients of these types of formulations [6,7]. To achieve a colon drug release, many matrix tablets are coated with polymers derived from methacrylic acid, known under the trade name Eudragit^®^, which are capable of forming a film that acts as a physical barrier between the medium and the core of the tablet, and they will react in the presence of specific conditions of the GIT modulating the rate of drug release. Thus, pH-dependent polymers such as Eudragit^®^ FS 30D respond to a change in the pH of the medium, while Eudragit^®^ RL 30D, a time-dependent polymer, responds to contact with the aqueous medium, forming a gelled layer that allows the sustained and controlled release of the drug, avoiding a burst effect [6]. Metronidazole is an antiprotozoal drug that is part of the nitrimidazole group, and it is frequently used as an antibiotic to treat a wide range of infections associated with anaerobic microorganisms. It is the first-choice antibiotic to treat colonic infections of *Clostridium difficile*, but it has also proven to be successful in the treatment of other colon pathologies involving anaerobic microorganisms, such as those present in uncomplicated diverticulitis disease [8].

The aim of this work was to use the ADAM module implemented in the Simcyp^®^ software program to predict biopharmaceutical and pharmacokinetic behaviors of the model drug, metronidazole, from hydrophilic matrices coated with different Eudragit^®^ polymers using the dissolution data obtained in vitro.

## 2. Materials and Methods

### 2.1. Design and Validation of the ADAM

The Simcyp^®^ Simulator (V18.1; Certara, Sheffield, UK) was used to build a metronidazole PBBM model employing the ADAM tool to predict drug behavior in vivo, using the physicochemical properties of metronidazole, experimental in vitro drug dissolution data, and population data compiled within the Simcyp^®^ software program. The ADAM implemented in Simcyp^®^ recreates the succession of events representing drug absorption in the human gastrointestinal tract, which is divided into nine anatomically well-differentiated segments from the stomach to the colon and through the small intestine. Drug absorption at each segment can be described as a function of release, depending on many different factors involving the physiology of the gastrointestinal tract (gastric emptying, transit times, luminal pH, fluid dynamics, gut wall enzymes and transporters, intestinal blood flow, food effect), drug-related factors (solubility, luminal degradation, permeability, gut wall metabolism, and gut wall transport), and formulation factors (drug particle size and formulation type). The ADAM module was run on the default population data of healthy volunteers in Simcyp^®^ following the design described in Table 1. This model was also used for validation and simulation. Sampling times were set at 200 points evenly distributed over the specified duration of the study [3].

The predictive performance of the PBBM biopharmaceutic model in ADAM was assessed by a numerical predictive check (NPC). For the validation of the PBBM model, plasma level data of metronidazole from pharmacokinetic studies using single-dose immediate-release (IR) dosage forms at a dose of 500 mg and sustained-release formulations of 750 mg (Flagyl ER), administered in a 7-day dosing regimen with a fasted 24 h dosing interval, were used [11]. Data on the in vitro release kinetics of sustained-release dosage forms of metronidazole (Flagyl ER) at a dose of 750 mg were also used for the validation procedure.

For validation, two populations with demographic characteristics and metronidazole dosage regimens similar to those used in the reference study populations were simulated using the ADAM module in Simcyp^®^ (Table 1) [9,10]. If observed concentrations from the literature for IR and ER references were distributed within the 90% PI, the model prediction capability was deemed to be adequate [12].

In addition, the overall predictability of the model was evaluated based on the average fold error (AFE). The calculated AFE was considered acceptable in the literature if it was within a 2-fold error (0.5–2-fold) [13]. The equation used for the calculation of AFE was the following:

$$AFE=10^{\frac{1}{n}\sum log(\frac{{PRED}_{Mean}}{OBS})}$$

### 2.2. Input Data

Metronidazole physicochemical properties (Table 2) were obtained from the literature. The blood-to-plasma partition ratio is predicted by the software using the values of plasma pH, hematocrit (%), and fu (fraction unbound in plasma). Metronidazole’s aqueous solubility is greatly affected by the pH of the medium. Metronidazole is a weak base ionizable at low pH values below its pKa, thereby making it soluble in aqueous medium conditions [14,15].

The effective permeability of metronidazole for the colon was introduced manually as reported in the literature [25]. Mean residence times (MRTs) at each segment of the gastrointestinal tract are calculated by the software considering the formulation as a monolithic controlled-release tablet (Table 3). Software predictions didn’t show sex differences for stomach and small intestinal MRTs; however, for the colon, there is a notable sex effect, whereby females have longer MRTs than males, which is considered in the model [3].

### 2.3. In Vitro Dissolution Profiles

ADAM simulations were carried out over the dissolution profiles of metronidazole-coated matrix tablets designed to achieve the maximum amount of dose into the colonic lumen. These formulations were based on hydroxypropyl methylcellulose (HPMC) and chitosan (CH) [26]. A 2^5^ fractional factorial design was performed considering formulation and process variables: polymer viscosity grade (HPMC K15/K35), polymer ratio (HPMC/chitosan 1:3 vs. 3:1), coating agent (Eudragit^®^ FS30D/Eudragit^®^ RL30D), blending time (10/20 min), and coating weight gain (10/20%). The study resulted in a total of 28 experimental batches, consisting of groups of 3 replicates and one batch without replicates, which represents the central point of the factors and levels tested. The dissolution profiles of the 28 batches were obtained following the methodology described in the previous publication [26].

**Figure 1:**
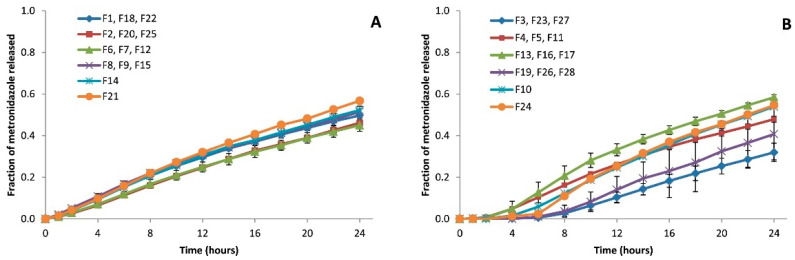
Mean release profiles from three replicates and all four central points. (**A**) Batches coated with Eudragit^®^ RL 30D (time-dependent coating); (**B**) Batches coated with Eudragit^®^ FS 30D (pH-dependent coating) [26].

The ADAM allows the introduction of the input dissolution data in three different ways: either using the Weibull function to fit the data, introducing the dissolution profiles simulating the gastric and small intestine environments directly into the platform, or inserting the solubility and dissolution data into the diffusion layer model function. In this work, dissolution profiles obtained in vitro from each developed batch (Figure 1) were introduced manually.

From the dissolution profiles, Simcyp^®^ calculates the dissolution rates using the linear method described by Yeh and Small [27].

### 2.4. Statistical Analysis

The normality of the distribution of the simulated pharmacokinetic data was assessed by the Shapiro–Wilk test using the Minitab^®^18 software program (Minitab LLC, State College, PA, USA).

The data were not normally distributed, and therefore, the statistical analysis was performed using the non-parametric Kruskal–Wallis statistical test. The factors considered to have an impact on pharmacokinetic responses were those with a *p* value ≤ 0.05. The factors studied were HPMC viscosity grade (mPas), HPMC/Chitosan ratio, blending time (min), coating agent, and % total weight increase after coating.

## 3. Results

### 3.1. Validation of the PBBM Model

The PBBM model developed allows the simulation of metronidazole plasma concentrations using different immediate and sustained release metronidazole dosage forms. These results were validated, showing a good predictive performance of the PBBM model used. Table 4 includes the mean observed and predicted values, and the fold errors corresponding to plasma concentrations of metronidazole using immediate release (IR) and extended release (ER) formulations.

**Figure 2:**
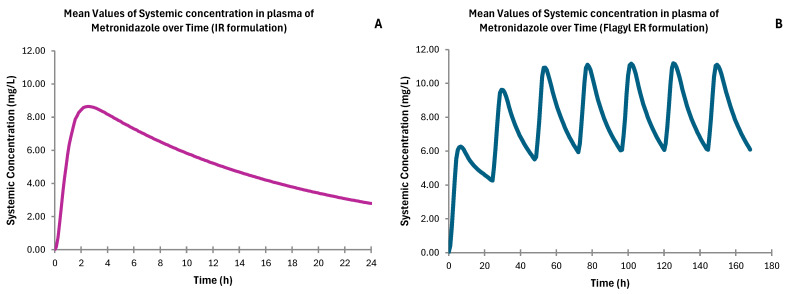
Simulated systemic plasma concentration profiles of metronidazole from the mean dissolution profiles for an IR tablet (**A**) and for Flagyl^®^ ER (**B**).

Figure 2 shows the systemic concentration of metronidazole over time from the reference formulations used for the model validation with pharmacokinetic results similar to values found in the literature, as can be seen in the fold error in Table 1.

**Figure 3:**
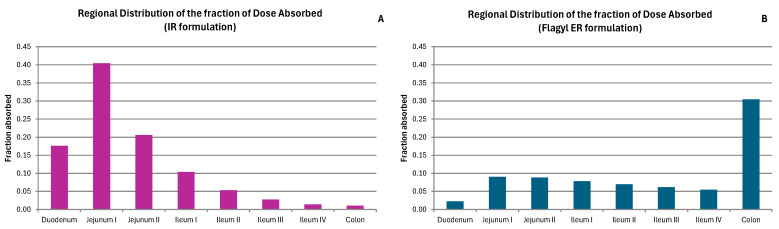
Regional distribution of the absorbed dose fraction of metronidazole simulated with the ADAM of an IR formulation (**A**) and a Flagyl^®^ ER formulation (**B**).

The ADAM allows us to discern which sections of the gastrointestinal system have absorption. Analyzing the fraction of absorbed dose in the different gastrointestinal segments, it can be deduced that for an immediate release formulation, the majority of the dose is absorbed in the small intestine. Metronidazole is absorbed in the middle segments of the small intestine, while the colon does not act as a main absorption site for the drug (Figure 3). In the case of metronidazole absorbed from the commercial ER formulation, the regional absorption pattern shows a higher proportion of dose absorbed in the colon, but there is a substantial fraction of dose absorbed in the rest of the gut (Figure 3).

### 3.2. Simulations of the PBPK Model

**Figure 4:**
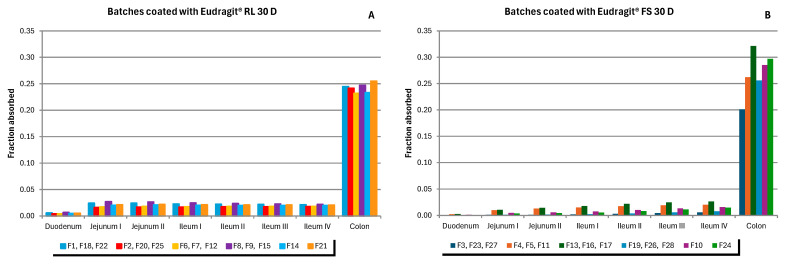
Regional distribution of the absorbed dose fraction of metronidazole simulated with the ADAM. Results are expressed as the average values out of every three batches developed under the same conditions and the values obtained from each of the four central points. (**A**) Batches coated with Eudragit^®^ RL 30D (time-dependent coating); (**B**) Batches coated with Eudragit^®^ FS 30D (pH-dependent coating).

Regarding the sustained-release colonic formulations of metronidazole evaluated in this study, although a difference is observed in the fraction absorbed of metronidazole from tablets coated with a pH-dependent polymer (FS 30D) compared to time-dependent coated tablets (RL 30D), both cases represent a very low fraction of metronidazole absorbed in comparison with an immediate release formulation, especially those coated with the pH-dependent polymer. Nevertheless, in a very small proportion, there is still a fraction of metronidazole absorbed from the batches developed. The colonic region, despite appearing to be another absorption site for metronidazole, has a much lower permeability than the upper parts of the gastrointestinal tract (Figure 4).

**Figure 5:**
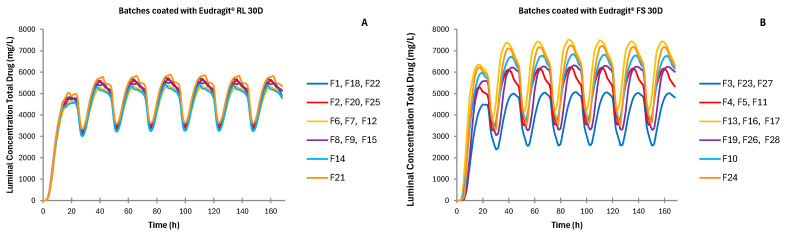
Simulated luminal concentration of metronidazole over time in the colon for batches coated with Eudragit^®^ RL 30D and batches coated with Eudragit^®^ FS 30D. (**A**) Batches coated with Eudragit^®^ RL 30D (time-dependent coating); (**B**) Batches coated with Eudragit^®^ FS 30D (pH-dependent coating).

Figure 5 shows the luminal concentration of metronidazole in the colon over time in the case of administering the formulations studied. Since the colon is less permeable to metronidazole and because there is a reduced amount of water in the colon, higher concentrations of the total drug are expected at this segment of the gastrointestinal tract.

**Figure 6:**
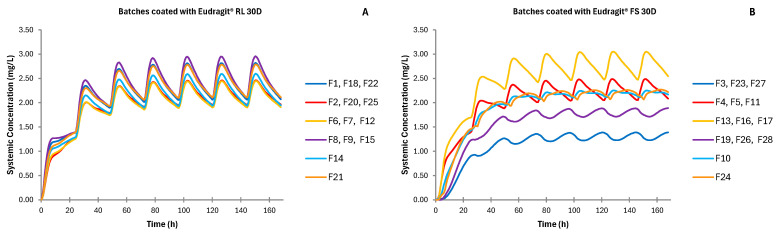
Simulated systemic concentration profiles out of the mean dissolution profiles of each of the three replicates and each central point for batches coated with Eudragit^®^ RL 30D (**A**) and for those coated with Eudragit^®^ FS 30D (**B**).

However, despite the lower systemic concentrations achieved from coated tablets, there is a clear difference between the systemic concentrations of metronidazole, depending on the coating agent employed. Figure 6 shows the mean values of systemic concentrations in plasma of metronidazole over time, considering the mean dissolution profiles obtained from all the batches studied. Tablets coated with Eudragit^®^ RL 30D show remarkable systemic drug concentration profiles with less variability than those obtained for batches coated with the pH-dependent polymer.

Average AUC, C_max_, and T_max_ values for each replicate group are shown in Table 5 and Table 6.

### 3.3. Statistical Analysis

The results of the Kruskal–Wallis statistical test were used to evaluate the effect of different formulation factors on the simulated pharmacokinetic parameters (AUC, Tmax, and C_max_). The H-values of the test and their corresponding *p*-values are shown. An asterisk (*) indicates statistically significant differences (*p* < 0.05) (Table 7).

The Kruskal–Wallis analysis demonstrates that the factors with a significant impact on the pharmacokinetic parameters include the percentage weight gain of the coating and the coating agent (Table 7).

## 4. Discussion

The ADAM implemented in Simcyp^®^ is a powerful tool that simulates the biopharmaceutical and pharmacokinetic behavior of oral drug delivery systems based on in vitro information and PBPK modeling and simulation. The simulations mainly reflect the behavior of the drug in the gastrointestinal tract. It should be noted that the results obtained in this study are preliminary; further in vivo studies are required to confirm these findings. The validation of the PBBM model used in this study demonstrated adequate predictive performance of the model using pharmacokinetic data for immediate- and sustained-release dosage forms.

The in vitro dissolution profiles used for the simulations show that tablets coated with the pH-dependent polymer have a latency time of up to 6 h, depending on the amount of polymer applied, in which the drug is not released into the medium [26]. As the total weight of the coating increases, drug release is delayed further because more time is required to dissolve the coating. This is subsequently reflected in the in vivo simulated pharmacokinetic behavior, which shows that the batches coated with an increase in total weight of 15–20% of Eudragit^®^ FS 30D reached a maximum systemic concentration after 6.6–14.1 h depending on the viscosity grade of the core polymer (Table 5 and Table 6). Batches coated with Eudragit^®^ RL 30D, however, did not show lag time periods, and drug release starts after the first hour. Time-dependent polymers act as an extra gel barrier between the medium and the tablet’s core, acting as the main controller of drug release [28]. Metronidazole is released into the environment when the tablet reaches the stomach, where the coating layer will gel alongside the tablet core. Metronidazole is a weak base easily dissolved in acidic mediums, and thus, it can be absorbed in the stomach. In addition, once the tablet reaches the small intestine, all metronidazole dissolved there will be absorbed [20,29], which explains the slightly higher metronidazole concentrations in the AUC results of these batches (Table 5 and Table 6, and Figure 6).

Batches manufactured with time-dependent coating show less variability than those with pH-dependent coating (Figure 4, Figure 5 and Figure 6). The combination of factors in the experimental design resulted in batches for the two types of coating with different coating thicknesses, but also with different types of tablet core [26]. These formulation differences resulted in different behaviors both in vitro and in vivo.

The colon has been observed to be the primary site of absorption for the Eudragit RL and FS formulations, contrasting with the behavior of the immediate-release formulations, which are predominantly absorbed in the proximal small intestine. As depicted in Figure 3A (IR formulation), the majority of absorption occurs in jejunum I and II, with less substantial absorption observed in the ileum and colon [30]. Figure 3B (Flagyl ER) shows a more uniform distribution of absorption across the entire gastrointestinal tract, with also a notable peak of absorption in the colon.

As demonstrated in Figure 4, the studied formulations exhibit a reduced fraction of absorbed dose in plasma when compared to the Flagyl^®^ ER formulation (Figure 3B). This observation indicates that the studied formulations could be more appropriate for achieving a local effect in the colon, promoting the release and retention of the drug in this region without significant absorption at the systemic level. This fact will contribute to potentially reducing the risk of adverse effects associated with the drug.

Once the tablets reach the colon, metronidazole accumulates in the colonic lumen (Figure 5) [31]. It is important to note that the permeability of metronidazole in the colon is rather low, and most of it is not absorbed [25], which justifies its accumulation in the lumen of the colon. The total concentration of the drug released in the lumen of the colon at 24 h is similar regardless of the type of matrix and coating employed, with higher variability for pH-dependent coated formulations. Still, different luminal concentration profiles are obtained depending on the coating agent used and the amount of coating applied. The simulated maximum concentration in the colonic lumen is reached a bit earlier and in a homogeneous way when tablets are coated with the time-dependent coating Eudragit^®^ RL 30D. This is because drug release has already begun in the upper parts of the gastrointestinal tract. Regardless of the composition of the tablet, high simulated concentration values of metronidazole in the colon can be achieved, far beyond the minimum inhibitory concentration (16 mg/L) for all the bacterial species affected by metronidazole [17,24]. Interestingly, the amount of metronidazole effective against microorganisms is maintained for longer periods (Figure 5). Currently, IR metronidazole formulation is prescribed twice a day, each for 12 h [32,33]. Increasing the luminal concentration residence time of metronidazole could lead to a change in administration, reducing dosage, and improving patient compliance.

The Kruskal–Wallis analysis shows how the percentage weight gain of the coating and the coating agent impact AUC, C_max_, and Tmax (Table 7). This could be explained by the different release mechanisms of the tested coating polymers because of its ability to control drug release differently.

## 5. Conclusions

PBBM models can help predict the release from drug delivery systems and the pharmacokinetics in vivo from data obtained in vitro although additional in vivo studies would be needed. In this work, we used the ADAM implemented in Simcyp^®^ to predict the pharmacokinetic behavior of coated matrix tablets of metronidazole designed to release their content at the colon. Different dissolution profiles were used depending on the type of coating agent used, namely Eudragit^®^ RL 30D and Eudragyt^®^ FS 30D, time- and pH-dependent polymers, respectively. pH-dependent coatings can delay drug release up to 6 h when the total weight gain of the coating is higher, which effectively avoids metronidazole release and its subsequent absorption in the upper parts of the gastrointestinal tract, thus reducing the appearance of potential adverse effects. Time-dependent coatings allow drug release within the first hour; however they act as an extra barrier, controlling release rates and avoiding an undesirable burst effect. Nevertheless, the amount of metronidazole released before the tablets reach the colon is rather low in both cases when compared to an immediate release formulation, pointing out that both polymers achieve effective drug release in the colonic region. The amount of metronidazole released in the colon can also vary depending on the percentage weight gain of the coating.

However, enough luminal concentration in the colon could be achieved with therapeutic effect over infections, despite the coating agent being used. The batches developed could potentially avoid metronidazole absorption in the upper parts of the gastrointestinal tract and thus reduce potential adverse effects associated with the treatment. Also, the batches developed could release a high enough concentration of metronidazole to treat infections in the colon at a pace that might lead to a reduction in the dosing regimen, which ultimately will improve patient treatment compliance. These findings with these metronidazole formulations should be confirmed in clinical trials with patients.

## Figures and Tables

**Table 1:** Trial design implemented in Simcyp^®^.

	IR Formulation *	Flagyl^®^ ER Formulation *	DoE Formulations **
Population Name	Healthy subjects	Healthy subjects	Sim-Healthy subjects
Population Size	9	24	1000
Number of Trials	1	1	100
No. of Subjects per Trial	9	24	10
Minimum years (age)	21	19	18
Maximum years (age)	23	46	65
Proportion of females	0.125	1	0.5
Study Duration (h)	24	168	168
Prandial State	Fasted	Fasted	Fasted
Route	Oral	Oral	Oral
Dose	500 mg	750 mg	500 mg
Dosing Regimen	Single Dose	Multiple Dose (7)	Multiple Dose (7)
Reference	[9]	[10]	-

* Experimental data used for model validation. ** Population simulated with Simcyp.

**Table 2:** Input parameter values used for the metronidazole simulations.

Parameter	Value	References/Comment
**Physico-chemical**		
Molecular weight (g/mol)	171.16	[16]
fu	0.8	[17]
log P_o:w_	−0.02	[18]
pKa	2.38	[19]
B/P	0.745	Predicted
Main plasma-binding protein	HSA	[20]
Aqueous solubility (mg/mL) *	10	[14,15]
**Absorption**		
Model	ADAM	
PSA (Å^2^)	83.9	[21]
HBD	1	[22]
fa	0.89	Predicted
Ka (h^−1^)	0.78	Predicted
Degradation rate constant (h^−1^)	2.58	[23]
**Distribution**		
Model	Minimal PBPK	
Vss (L/kg)	0.60	[24]
**Elimination**		
CL (L/h)	3.80	[24]

fu = fraction of drug unbounded in plasma; P_o:w_ = oil/water partition coefficient; B/P = blood-to-plasma partition ratio; HSA = human serum albumin; ADAM = Advanced Dissolution Absorption Model; PSA = polar surface area; HBD = hydrogen bond donors; fa = fraction available from dosage form; Ka = first-order absorption rate constant; Vss = steady state volume of distribution; CL = In vivo elimination clearance. * Aqueous solubility in water at 25 °C.

**Table 3:** Mean residence times (MRTs) for monolithic controlled release coated tablets at each segment of the gastrointestinal tract.

Organ		MRT (h)	CV (%)
Stomach		1.21	143.38
Small intestine		5.43	50.53
Colon	Male	29.14	83
	Female	41	83

**Table 4:** Mean values simulated and predictability of the model.

Reference Formulation	PK Parameters	Mean Value Observed	Mean Value Predicted ADAM	AFE *	Ref.
IR 500 mg	C_max_ (mg/L)	9.0 ± 0.5	8.7 ± 2.6	0.97	[9]
	T_max_ (h)	1.9 ± 0.2	2.6± 0.3	1.38	
	AUC (mg.h/L)	122.2 ± 10.3	126.2 ± 30.8	1.03	
Flagyl ER 750 mg	C_max_ (mg/L)	12.5 ± 4.8	11.4 ± 2.7	0.91	[10]
	T_max_ (h)	6.8 ± 2.8	5.6 ± 1.6	0.82	
	AUC (mg.h/L)	198.0 ± 75.3	202.7 ± 56.4	1.02	

* Average fold error.

**Table 5:** Mean pharmacokinetic parameters of metronidazole (AUC, C_max_, T_max_) simulated for the time-dependent batches. The results are expressed as the average values ± standard deviation out of every three batches developed in the same conditions and the values obtained from each of the four central points.

	BATCH	AUC (mg/L·h) (SD)	C_max_ (mg/L) (SD)	T_max_ (h) (SD)
Time-dependent coated tablets	F1	58.39 ± 20.24	2.91 ± 0.93	6.34 ± 2.33
	F18	58.37 ± 19.93	2.93 ± 0.92	6.37 ± 2.25
	F22	58.81 ± 19.99	2.98 ± 0.93	6.30 ± 2.15
	F2	54.19 ± 19.21	2.64 ± 0.88	6.59 ± 2.64
	F20	52.33 ± 18.53	2.55 ± 0.85	6.71 ± 2.70
	F25	52.05 ± 18.90	2.53 ± 0.87	6.84 ± 2.85
	F6	53.86 ± 18.72	2.67 ± 0.87	6.56 ± 2.48
	F7	54.53 ± 18.86	2.68 ± 0.87	6.52 ± 2.47
	F12	47.75 ± 17.28	2.32 ± 0.79	6.75 ± 2.80
	F8	64.47 ± 22.09	3.30 ± 1.03	6.14 ± 2.00
	F9	58.94 ± 20.82	2.99 ±0.97	6.24 ± 2.10
	F15	58.18 ± 20.33	2.92 ± 0.94	6.37 ± 2.25
	F14	54.33 ± 19.02	2.71 ± 0.88	6.42 ± 2.34
	F21	58.64 ± 20.36	2.91 ± 0.94	6.42 ± 2.33

**Table 6:** Mean pharmacokinetic parameters of metronidazole (AUC, C_max_, T_max_) simulated for the pH-dependent batches. Results are expressed as the average values ± standard deviation out of every three batches developed in the same conditions and the values obtained from each of the four central points.

	BATCH	AUC (mg/L·h) (SD)	C_max_ (mg/L) (SD)	T_max_ (h) (SD)
pH-dependent coated tablets	F3	29.17 ± 11.68	1.33 ± 0.51	7.22 ± 7.90
	F23	33.66 ± 13.26	1.54 ± 0.58	7.92 ± 9.02
	F27	39.10 ± 15.28	1.77 ± 0.66	8.64 ± 8.43
	F4	52.79 ± 18.96	2.51 ± 0.87	7.90 ± 3.89
	F5	56.26 ± 19.90	2.70 ± 0.92	7.67 ± 3.64
	F11	55.17 ± 19.38	2.65 ± 0.90	8.23 ± 4.00
	F13	63.84 ± 24.26	2.97 ± 1.11	10.72 ± 5.44
	F16	69.23 ± 25.14	3.48 ± 1.21	7.31 ± 2.97
	F17	67.66 ± 23.46	3.22 ± 1.10	10.93 ± 5.17
	F19	27.94 ± 11.49	1.29 ± 0.50	6.56 ± 8.25
	F26	47.12 ± 18.84	2.14 ± 0.82	8.25 ± 8.89
	F28	54.68 ± 22.00	2.47 ± 0.96	10.46 ± 9.14
	F10	52.81 ± 20.37	2.41 ± 0.90	10.53 ± 6.65
	F24	53.05 ± 21.00	2.41 ± 0.93	14.07 ± 8.12

**Table 7:** Kruskal–Wallis results for pharmacokinetic parameters studied. %ΔW = % increase on total weight; Ratio HPMC/Chi: HPMC/Chitosan ratio; * *p* < 0.05. n = 14 for each group.

Response	Factors (Groups)	H	*p* Value
AUC	Coating agent	1.54	0.215
	%ΔW	16.77	<0.001 *
	HPMC	0.84	0.358
	Ratio HPMC/Chi	1.48	0.476
	Blending time	0.67	0.714
C_max_	Coating agent	4.47	0.035 *
	%ΔW	15.92	<0.001 *
	HPMC viscosity grade	1.22	0.270
	Ratio HPMC/Chi	0.95	0.621
	Blending time	1.04	0.593
T_max_	Coating agent	18.65	<0.001 *
	%ΔW	1.42	0.493
	HPMC viscosity grade	0.03	0.854
	Ratio HPMC/Chi	0.47	0.790
	Blending time	0.43	0.805

## Data Availability

The data presented in this study are available within the article.
